# Prolonged Emesis Causing Esophageal Perforation: A Case Report

**DOI:** 10.7759/cureus.24720

**Published:** 2022-05-04

**Authors:** Nandita Kakar, Harrison C Smith, Anthony M Shadid

**Affiliations:** 1 Medicine, Nova Southeastern University Dr. Kiran C. Patel College Of Osteopathic Medicine, Fort Lauderdale, USA; 2 Radiology, University of Illinois at Chicago, Peoria, USA

**Keywords:** emesis, crepitus, subcutaneous emphysema, esophageal perforation, boerhaave syndrome

## Abstract

Transmural esophageal rupture or Boerhaave syndrome carries a high mortality rate due to delayed diagnosis and treatment. The heterogeneity of symptoms, age, comorbidities, and the severity of illness in this group of patients add to the difficulty of the management of Boerhaave syndrome. It generally occurs in the distal part of the esophagus and may result in the leakage of gastric contents into the thoracic cavity leading to mediastinal necrosis and bacterial infection. The management relies on prompt detection and intervention with conservative care and/or surgical repair. Early recognition within 24 hours followed by primary repair of the esophagus with mediastinal and chest drainage is associated with a 90% survival rate.

## Introduction

Boerhaave syndrome, also known as a spontaneous esophageal tear due to an acute increase in intraesophageal pressure, has an incidence rate of 3.1 per one million people per year. While its most common cause involves forceful emesis, the condition can also occur in the setting of weight lifting, defecation, childbirth, or other causes of increased pressure in the esophagus. Symptoms may vary and present with coexisting medical conditions, which can make the diagnosis challenging. Delays in treatment can prove to be fatal with mortality rates of up to 40%, and the absence of treatment is associated with mortality rates of up to 90% [1]. We present a case of Boerhaave syndrome that was managed with conservative care thanks to a timely and prompt diagnosis.

## Case presentation

A 24-year-old female with a past medical history of acute pancreatitis presented to the emergency department with acute-onset retrosternal chest pain after numerous emetic episodes for about 10 hours following excessive alcohol intake for the past four days. She also reported three episodes of diarrhea and sharp epigastric abdominal pain. She denied any urinary symptoms and reported that her last menses had ended one week ago and had been normal. She denied any current medications and reported an alcohol intake of about one glass of wine a day and marijuana use about once a week. The patient stated that her history of acute pancreatitis was secondary to binge drinking.

Physical examination revealed a well-developed, anxious patient with dry mucous membranes, mild diffuse tenderness and crepitus upon chest palpation, and mild epigastric abdominal tenderness upon palpation with no guarding, rigidity, or rebound tenderness of the abdomen. The patient had a BMI of 24.8 kg/m^2^ and her vital signs included a temperature of 98.6 °F, blood pressure of 127/74 mmHg, pulse rate of 123 bpm, respiratory rate of 13/minute, and oxygen saturation of 98%.

**Figure 1:**
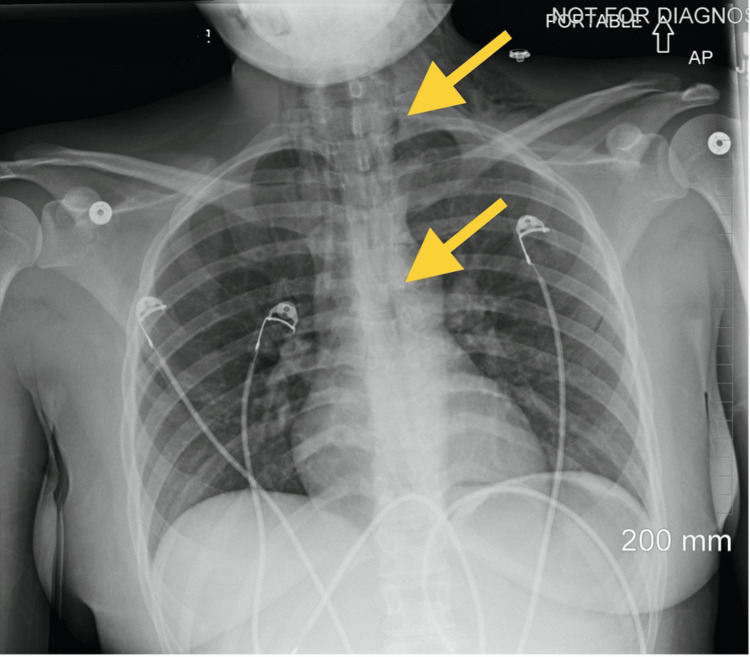
Chest X-ray showing pneumomediastinum and subcutaneous emphysema at the cervicothoracic junction (arrows)

Lab results revealed no significant findings and all values for complete blood count, comprehensive metabolic panel, and lipase were within normal range. A chest X-ray was obtained and revealed mildly decreased lung volume bilaterally with no evidence of lobar collapse, pulmonary edema, or pleural effusion. However, there was upper pneumomediastinum and subcutaneous emphysema centered at the cervicothoracic junction with more prominent air seen in the left supraclavicular region (Figure 1). This combination limited the evaluation of the lung apices, but taking this into account, no definite pneumothorax was seen. These findings raised suspicion for esophageal perforation and further evaluation with a CT of the neck and chest with the administration of oral contrast was recommended.

**Figure 2:**
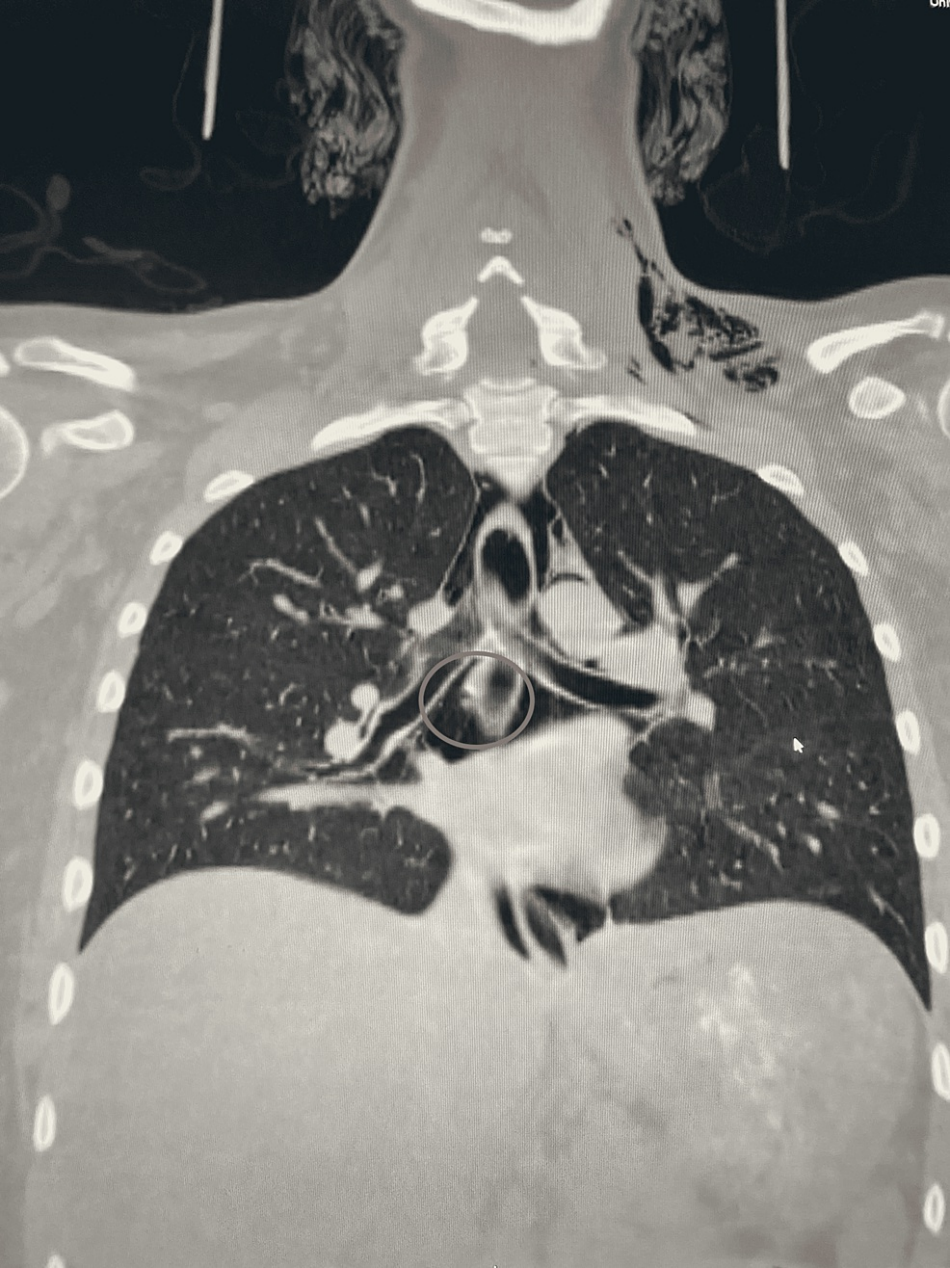
CT scan of head and neck The image shows a 2-mm tear of the right mid esophagus with extensive pneumomediastinum and subcutaneous gas at the base of the neck CT: computed tomography

CT scan of the neck and chest was performed utilizing effervescent crystals and oral contrast (Figure 2). Extensive subcutaneous emphysema extending from the base of the skull inferiorly and bilaterally into the mediastinum was observed. Mild gas distension and enteric contrast were seen within the esophagus. There was a focus of wall irregularity and focus of intramural contrast noted in the right esophageal wall just below the carina. There was also a 2-mm tear of the esophageal wall seen on coronal images. There was no extravasation of enteric contrast into the mediastinum or the neck. No pericardial effusion or pericardial gas was visualized in the mediastinum as well as no evidence of mediastinal hematoma, abscess, or fluid collection. The lungs revealed no pneumothorax, pleural effusion, consolidation, or bronchiectasis.

**Figure 3:**
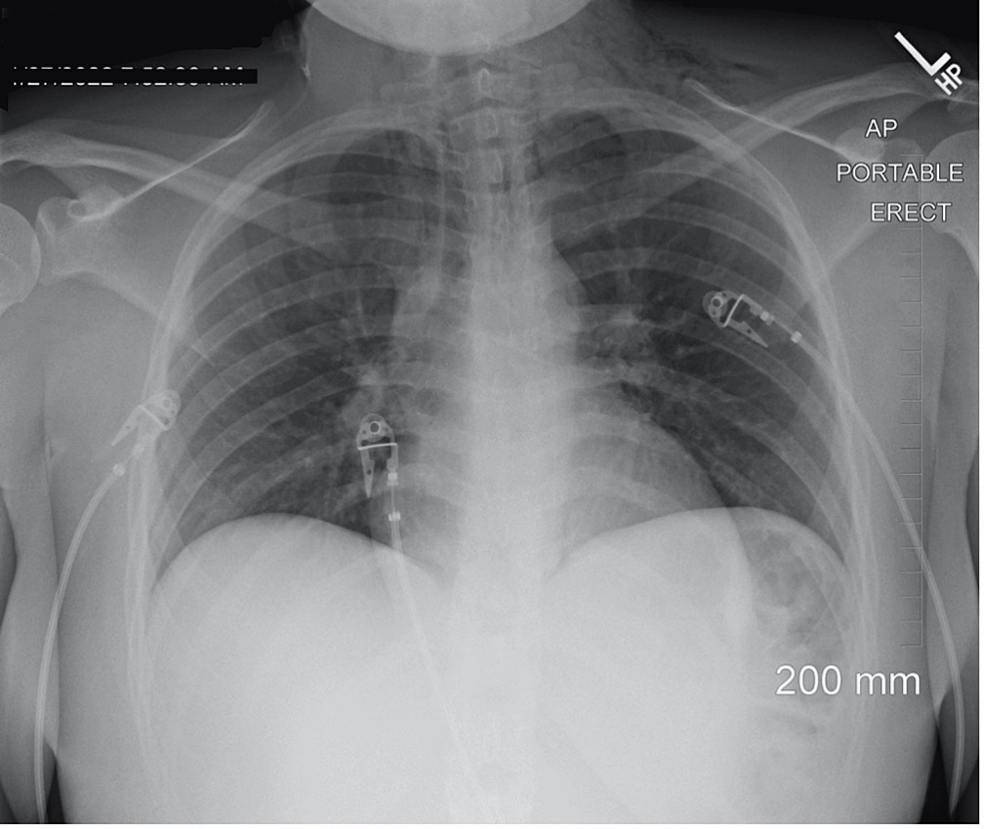
Repeat chest X-ray showing the resolution of free air in the mediastinum and cervicothoracic junction

The patient was admitted and placed under close observation overnight and was started on intravenous fluids, proton pump inhibitors (PPI), broad-spectrum antibiotics, imipenem/cilastatin, and was ordered to receive nothing by mouth (NPO). Within 12 hours, the patient’s pain and dyspnea started to improve. A repeat chest X-ray was performed and compared to the previous X-ray imaging (Figure 3). There was a marked resolution of free air in the thoracic cavity and soft tissue spaces. Surgical intervention was deferred and the patient was continued to be monitored under conservative care. She was discharged five days later following supportive care with instructions for a soft diet for one week and follow-up with primary care. The patient was also educated on alcohol consumption and abuse.

## Discussion

Boerhaave syndrome is reported to have a male predominance with a male-to-female ratio ranging from 2:1 to 5:1 and is more frequently seen in people aged 50-70 years. It is caused by an abrupt increase in intraesophageal pressure leading to a transmural tear through the esophagus. These ruptures are usually longitudinal, most commonly occurring in the distal esophagus, about 2-4 cm above the cardia. A higher incidence rate has been reported in the left posterolateral wall, thought to be caused by anatomical weakness likely due to the large presence of vascular and neural structures [2]. The patient in this case developed Boerhaave syndrome in the distal right esophagus due to prolonged retching after excessive alcohol intake. Forceful emesis only accounts for 15-30% of esophageal perforations and the rest results from iatrogenic, traumatic, foreign-body, and pathological perforations. Ruptures in the distal esophagus may result in the spillage of gastric contents leading to widespread mediastinal and pleural inflammation, emphysema, and necrosis. These complications may lead to acute respiratory distress syndrome (ARDS) and even death [2].

The presence of the Mackler’s triad of vomiting, chest pain, and subcutaneous emphysema, and other associated symptoms like tachycardia, fever, tachypnea, and epigastric pain aid in the diagnosis of Boerhaave syndrome. However, these symptoms are not specific to esophageal perforation and can also be seen in conditions like peptic ulcer disease, acute pancreatitis, and myocardial infarction. Delays in diagnosis increase the rate of complications associated with esophageal rupture. Boerhaave syndrome is known as a lethal gastrointestinal tract disorder with mortality rates of up to 40% [1]. This condition can be diagnosed with prompt radiological studies including contrast esophagram with Gastrografin, chest X-ray, and CT scan of the chest and abdomen. Gastrografin is a water-soluble contrast that is used in real-time fluoroscopy as a first-line option for the diagnosis of Boerhaave syndrome. Gastrografin contrast is preferred over barium contrast to avoid mediastinitis and the long-term effects of barium in the pleural space [3]. However, if there is high suspicion and the test is negative, it should be followed by a barium esophagram or CT scan for confirmation [4]. CT scan is the preferred diagnostic imaging modality due to its better diagnostic specificity, but chest X-rays are also found to be abnormal in 90% of cases [4].

The management is tailored to the patient’s presentation, the extent of the rupture, the time since the rupture, and its associated complications. The common treatment options include conservative, endoscopic, or surgical. The mainstay of all treatment includes PPI, intravenous fluids, broad-spectrum antibiotic coverage, NPO, and surgical evaluation [5]. The patient in this case presented to the emergency department immediately after the acute onset of chest pain, and esophageal rupture was high on the differential due to the patient’s 10-hour history of intense vomiting and crepitus upon chest palpation. The abnormal X-ray findings led to further CT assessment, which revealed a small tear in the right distal esophagus. The finding of a transmural esophageal tear raised concern for a plethora of potential complications. Surgical consultation was made immediately for laparoscopic primary repair in the event of a decline in patient condition. The lack of evidence for mediastinal or pleural contamination was a positive prognostic factor for this patient. The perforation was considered contained and managed with PPI therapy, nasogastric suction, and NPO status to prevent exacerbating gastric activity. Broad-spectrum antibiotics were also administered to prevent aspiration pneumonia or other complications of microbial invasion that may be caused by further exacerbations or fluid collection that may have been too small to be seen on imaging. Imipenem/cilastatin was the antibiotic of choice for anaerobic and aerobic gram-positive as well as gram-negative bacteria including Pseudomonas and Enterococcus.

Repeat chest X-ray imaging showed marked resolution of free air in the thoracic cavity. There was still no evidence of mediastinal or pleural fluid collection. The patient did not require surgical intervention for chest tube placement or laparoscopic repair because the esophageal tear was contained within the mediastinum, few symptoms were present, and the patient showed no signs of sepsis. The timely and prompt diagnosis may have played a part in preventing life-threatening conditions in this patient.

Surgical intervention is advocated for larger tears and gastric content leakage within 24 hours to prevent complications caused by friable and edematous wound edges. This procedure involves the direct repair of the rupture complemented by adequate drainage of the mediastinum and pleural cavity [6]. Nutritional supplementation with feeding gastrostomy is required in patients with prolonged NPO status. Endoscopic placement of esophageal stents is also used to seal esophageal leaks as well as to prevent fistula formations. Perforations diagnosed within 12-24 hours are associated with a successful outcome rate of 90% [4].

Outcomes of direct repair of esophageal perforations accompanied by complications are highly variable depending on individual health status. A damage-control approach involves two steps in which the patient is first subjected to tube thoracostomy drainage and debridement of the mediastinum and pleural cavity to stabilize the patient and gain sepsis control. Direct esophageal repair is followed in a subsequent surgery. This approach may require two thoracotomies and an increased length of hospital stay but is shown to reduce morbidity and mortality in certain cases [2].

## Conclusions

The concept of the "golden period" of the first 24 hours applies to the diagnosis and treatment of esophageal perforation. Prompt recognition and management are critical and have been shown to decrease mortality rates in this group of patients. The most common symptoms include vomiting, lower thoracic pain, epigastric pain, and pulmonary signs such as subcutaneous emphysema. Primary laparoscopic repair is the mainstay of treatment; however, a damage-control approach may also be taken to control sepsis with thoracic drainage before esophageal repair. Conservative care monitored with repeat imaging may also be employed as the treatment of choice for patients with few symptoms and esophageal tears that are contained, as seen in this case.
